# Administration of a tropomyosin receptor kinase inhibitor attenuates sarcoma-induced nerve sprouting, neuroma formation and bone cancer pain

**DOI:** 10.1186/1744-8069-6-87

**Published:** 2010-12-07

**Authors:** Joseph R Ghilardi, Katie T Freeman, Juan M Jimenez-Andrade, William G Mantyh, Aaron P Bloom, Michael A Kuskowski, Patrick W Mantyh

**Affiliations:** 1 Research Service, VA Medical Center, One Veterans Drive, Minneapolis, MN 55417, USA; 2 Department of Pharmacology, University of Arizona, 1656 E. Mabel, Tucson, AZ 85724, USA; 3 GRECC, VA Medical Center, One Veterans Drive, Minneapolis, MN 55417, USA; 4 Arizona Cancer Center, University of Arizona, 1656 E. Mabel, Tucson, AZ 85724, USA

## Abstract

Pain often accompanies cancer and most current therapies for treating cancer pain have significant unwanted side effects. Targeting nerve growth factor (NGF) or its cognate receptor tropomyosin receptor kinase A (TrkA) has become an attractive target for attenuating chronic pain.

In the present report, we use a mouse model of bone cancer pain and examine whether oral administration of a selective small molecule Trk inhibitor (ARRY-470, which blocks TrkA, TrkB and TrkC kinase activity at low nm concentrations) has a significant effect on cancer-induced pain behaviors, tumor-induced remodeling of sensory nerve fibers, tumor growth and tumor-induced bone remodeling. Early/sustained (initiated day 6 post cancer cell injection), but not late/acute (initiated day 18 post cancer cell injection) administration of ARRY-470 markedly attenuated bone cancer pain and significantly blocked the ectopic sprouting of sensory nerve fibers and the formation of neuroma-like structures in the tumor bearing bone, but did not have a significant effect on tumor growth or bone remodeling.

These data suggest that, like therapies that target the cancer itself, the earlier that the blockade of TrkA occurs, the more effective the control of cancer pain and the tumor-induced remodeling of sensory nerve fibers. Developing targeted therapies that relieve cancer pain without the side effects of current analgesics has the potential to significantly improve the quality of life and functional status of cancer patients.

## Background

Cancer pain can have a significant impact on the quality of life and functional status of the individual [1,2]. A major reason cancer pain remains a significant health problem is the limited repertoire and negative side effects of currently available analgesics. For example, non-steroidal anti-inflammatory drugs, which are effective in reducing a variety of musculoskeletal pains, have been shown to have significant gastrointestinal side effects [3,4]. Opiates are also frequently used to treat moderate to severe cancer pain. While opiates are highly effective at controlling ongoing cancer pain, as a class opiates have a variety of unwanted side effects including increased somnolence, agitation, constipation, dizziness, cognitive impairment and respiratory depression [5,6].

**Figure 1:**
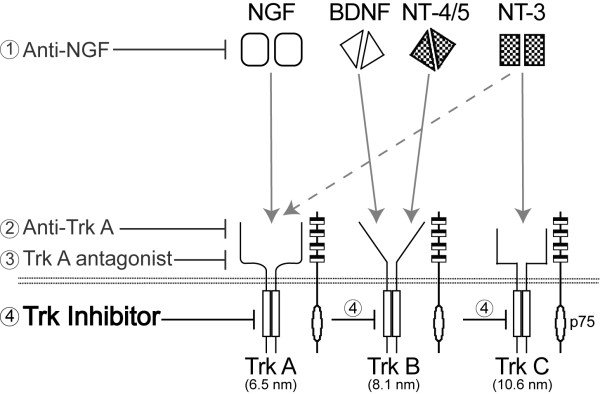
**Strategies for targeting NGF/TrkA for pain relief**. Current strategies for targeting NGF or its cognate receptor TrkA include; monoclonal antibodies or peptibodies that sequester NGF (1), monoclonal antibodies that target TrkA and prevent NGF from binding to TrkA (2), small molecule TrkA antagonist therapy (3) and the focus of the current study, a small molecule kinase inhibitor of Trk's (4). The Trk inhibitor used in this study (ARRY-470) is a small molecule inhibitor demonstrating nanomolar cellular inhibition of TrkA (6.5 nM), TrkB (8.1 nM), and TrkC (10.6 nM) and a high level of selectivity over a panel of kinase and non-kinase receptors (Additional file 1 Table S2 and S3). Schematic drawing adapted from Pezet and McMahon [17].

Recently, peripherally restricted targeting of nerve growth factor (NGF) or its cognate tropomyosin receptor kinase A (TrkA) has become an attractive target for attenuating chronic pain. Four major strategies are currently being pursued (Figure 1) and each of these strategies has its potential strengths and limitations [7,8]. For example, while monoclonal antibodies (mAbs) are extraordinarily specific in their targeting, administration of mAbs carries the risk of immune reactions such as acute anaphylaxis, serum sickness and the generation of antibodies against the therapeutic agent. In contrast, small molecule inhibitors of kinase activity do not require intravenous or intramuscular injection, are generally less expensive to make than mAbs, allow greater flexibility in dosing, but are generally less selective than mAbs [8]. Whether the kinases' lack of extraordinary specificity found with mAbs will provide greater desired efficacy or greater unwanted side effects will probably need to be examined with each mAb or kinase(s) that is being targeted.

In the present paper we use a mouse model of bone cancer pain to demonstrate that early administration of a small molecule kinase Trk inhibitor, ARRY-470, significantly reduces cancer pain in the early, middle and late time points in disease progression. Interestingly, the cancer and its associated stromal cells induced a remarkable sprouting and neuroma formation by sensory nerve fibers that innervate the tumor-bearing bone and this sprouting and neuroma formation was markedly attenuated by Trk inhibition. In contrast, Trk inhibition had no significant effect on tumor growth or tumor-induced bone remodeling in this model.

## Results

### Early, but not late ARRY-470 administration significantly attenuates tumor-induced pain

**Figure 2:**
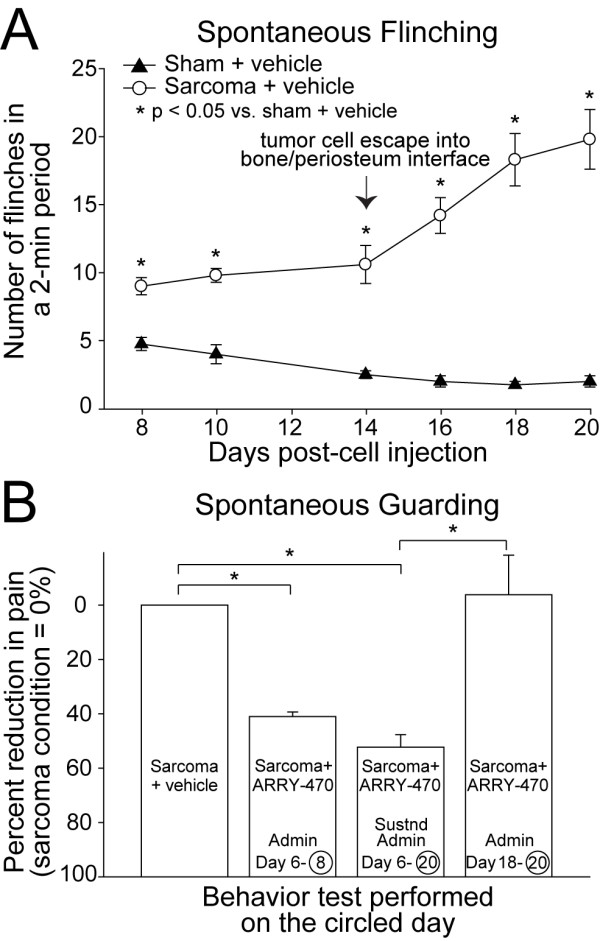
**ARRY-470 can significantly reduce bone cancer pain behaviors**. Injection of green fluorescent protein (GFP)^+ ^sarcoma cells into the intramedullary space of the femur results in significantly greater spontaneous flinching pain behaviors compared to sham injected mice from day 8 until day 20 post cell injection (**A**). Note that at day 14 there is a rapid escalation of pain behaviors, which is when invasion and growth of GFP^+ ^cancer cells occurs within the periosteum. ARRY-470 therapy significantly reduces cancer pain behaviors if this therapy is administered before sprouting and neuroma formation occur, in either an early/acute (days 6-8), or an early/sustained (days 6-20) fashion (**B**). In contrast, ARRY-470 administered at late time points (days 18-20), when nerve sprouting and neuroma-like structures have already formed, did not significantly reduce cancer-related spontaneous guarding pain behaviors. Each point or bar represents the mean ± SEM. Brackets indicate the groups being compared. *p < 0.01.

To assess whether inhibition of the Trks attenuates bone cancer pain, pain behaviors were analyzed in sham + vehicle and in tumor-bearing mice treated with early/acute ARRY-470, early/sustained ARRY-470, and late/acute ARRY-470. These behavioral analyses confirmed previous observations [9] that at early time points (days 8-14 post tumor cell injection), pain-related behaviors gradually increase in severity with time (Figure 2A), and correlate with tumor growth in the intramedullary space of the femur, as well as progressive tumor-induced bone destruction. Interestingly, pain behaviors escalated rapidly upon the escape of sarcoma cells from the intramedullary space (days 14-20 post tumor injection) (Figure 2A), which resulted in tumor-induced sprouting of CGRP^+ ^and NF200^+ ^nerve fibers in the periosteum. Behavioral analysis revealed that when ARRY-470 was given from days 6-8 post tumor injection, pain behaviors were reduced by ~40% at day 8, whereas early/sustained administration of ARRY-470 from days 6-20 reduced pain behaviors by ~60% at day 20. In contrast, late administration of ARRY-470 (initiated at day 18-20) did not significantly reduce cancer pain behaviors by day 20 (Figure 2B).

### Tumor growth induces profuse sprouting of sensory nerve fibers and the formation of neuroma-like structures

**Figure 3:**
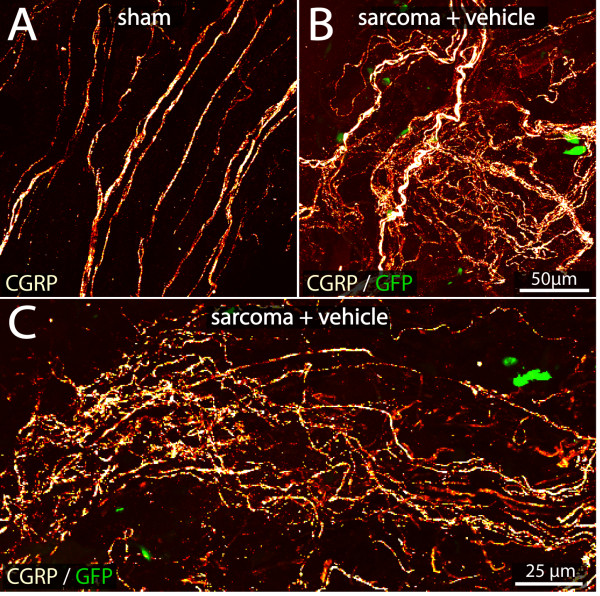
**Sprouting of sensory nerve fibers in the tumor bearing bone**. Confocal images of non-decalcified whole mount preparations of the femoral periosteum from sham (A) or sarcoma + vehicle mice (B, C) immunostained with calcitonin gene-related peptide (CGRP), a marker of peptide-rich C-fibers and some A-delta sensory nerve fibers. Note the increased density and disorganized appearance of CGRP^+ ^nerve fibers (in red) in the periosteum of the tumor-injected femur (B, C) compared to the periosteum of sham animals (A). This pathological sprouting pattern is found in the periosteum near viable tumor/stromal cells (C). Confocal images of periosteum were acquired from whole mount preparations and projected from 280 optical sections at 0.25 μm intervals with a 40x objective.

Tumor-induced changes were examined in the periosteum, as this bone compartment is richly innervated by sensory nerve fibers [10], appears to be pivotally involved in detecting injury to the skeleton [11], and is the only tissue in bone that can be immunohistochemically analyzed in both decalcified frozen sections and non-decalcified whole mount preparations [10]. In naïve and sham + vehicle-treated animals CGRP^+ ^sensory nerve fibers have a net-like organization and are typically associated with blood vessels. In sham mice, there was no difference in the organization or density of CGRP^+ ^(Figure 3A) periosteal nerve fibers compared to naïve mice (data not shown). Twenty days following tumor cell injection, we observed significant sprouting by CGRP^+ ^and NF200^+ ^sensory nerve fibers. These fibers appeared to be intermingled among GFP^+ ^tumor cells, and had a disorganized appearance that is never observed in the periosteum of naïve or sham mice.

**Figure 4:**
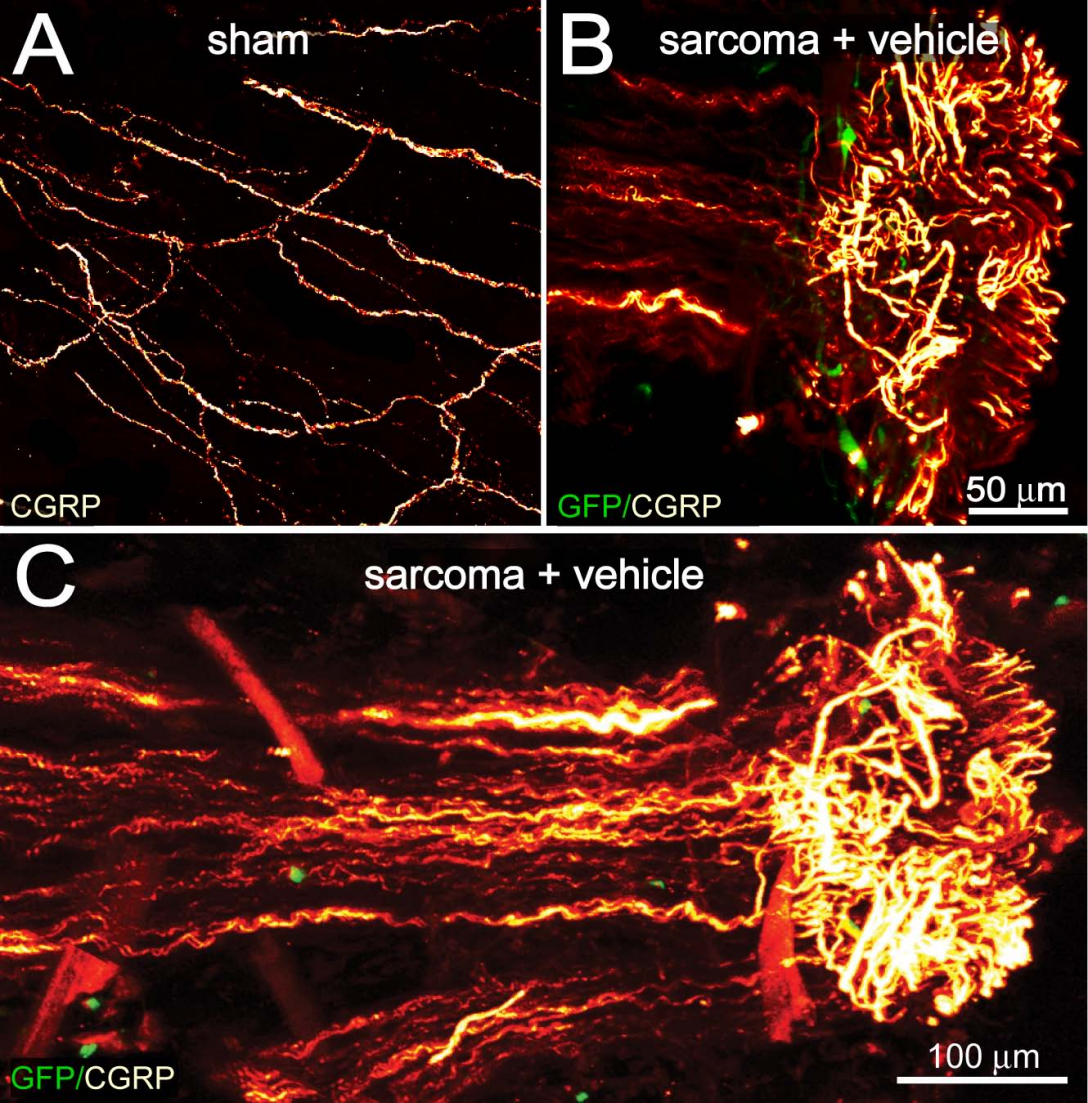
**Tumor-induced formation of neuroma-like structures by sensory nerve fibers**. Confocal images of non-decalcified whole mount preparations of the femoral periosteum from sham (A) or sarcoma + vehicle mice (B, C) immunostained with calcitonin gene-related peptide (CGRP). Note this mass of nerve fibers has the hallmark of a neuroma-like structure i.e. mass of disordered, blind ending axons that have an interlacing or whirling morphology (in red) and these neuroma-like formations are only observed in the periosteum of the tumor-injected femur (B, C) as compared to the periosteum of sham animals (A). Confocal images of periosteum (approximately 70 μm in thickness) were acquired from whole mount preparations and projected from 280 optical sections at 0.25 μm intervals with a 40x objective.

**Figure 5:**
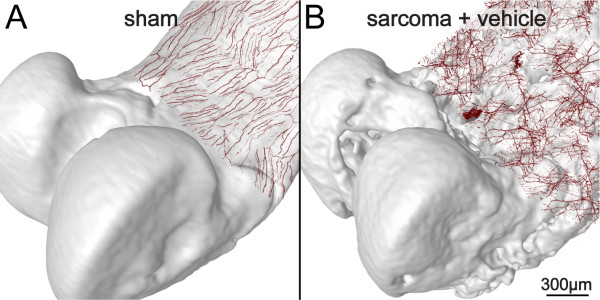
**Overlay of confocal images of whole mount preparations onto μCT images of bone showing sprouting and neuroma formation by sensory nerve fibers**. Confocal images of whole mount preparations of the femoral periosteum from sham (A) or sarcoma + vehicle mice (B) immunostained with calcitonin gene-related peptide (CGRP) and overlayed onto uCT images of the bones from which they were obtained. Confocal images of periosteum (approximately 70 μm in thickness) were acquired from whole mount preparations and projected from 280 optical sections at 0.25 μm intervals with a 40x objective. Z-stack images of whole mount preparations from four sham or sarcoma + vehicle mice were acquired, tiled, and overlaid to scale on a three-dimensional micro-CT image of the sham femur (A) and sarcoma + vehicle femur (B), respectively using AMARA software. Note that the tumor-injected femur (B) has severe cortical bone deterioration and a pathological reorganization of CGRP nerve fibers (in red) compared to the sham femur (A).

Whereas all mice with GFP^+ ^tumor cells growing in the periosteum showed significant sprouting of CGRP^+ ^and NF200^+ ^nerve fibers, approximately one half of these mice had 1-2 neuroma-like structures in the periosteum (Figure 4B, Figure 5B). These neuroma-like structures appear as a disordered mass of blind ending axons that have an interlacing or whirling morphology [12,13] and are never observed in sham or naïve animals.

### Early blockade of Trk's attenuates tumor-induced nerve sprouting and formation of neuroma-like structures and does not significantly affect disease progression

**Figure 6:**
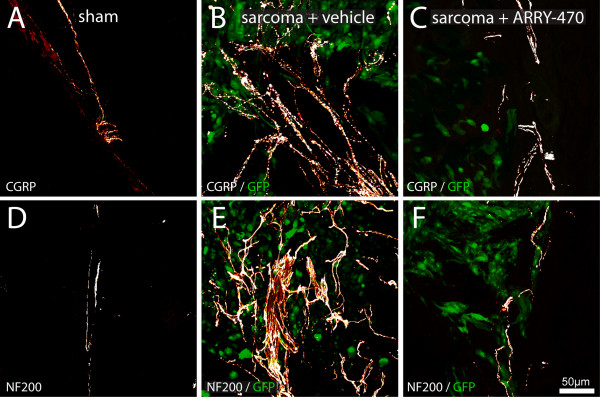
**Preventive Trk inhibition reduces CGRP^+ ^and NF200^+ ^nerve fiber sprouting and the formation of neuroma-like structures in the periosteum of tumor-injected mice**. Representative confocal images of periosteum from sham (A, D), sarcoma + vehicle (B, E), and sarcoma + early/sustained ARRY-470 (C, F) mice. Periosteum sections were immunostained with an antibody against CGRP (A-C) and NF200 (D-F). Note that at day 20 post-tumor cell injection there is significant sprouting and neuroma formation by CGRP^+ ^(B) and NF200^+ ^(E) nerve fibers in sarcoma + vehicle mice. Preventive and maintained administration of ARRY-470 **(**30 mg/kg; p.o., BID) initiated at day 6 through day 20 post cell injection significantly reduces the pathological tumor-induced reorganization of sensory CGRP^+ ^(C) and NF200^+ ^(F) nerve fibers. Confocal images were acquired from bone sections (20 μm in thickness) and were projected from 80 optical sections at 0.25 μm intervals with a 40x objective.

**Figure 7:**
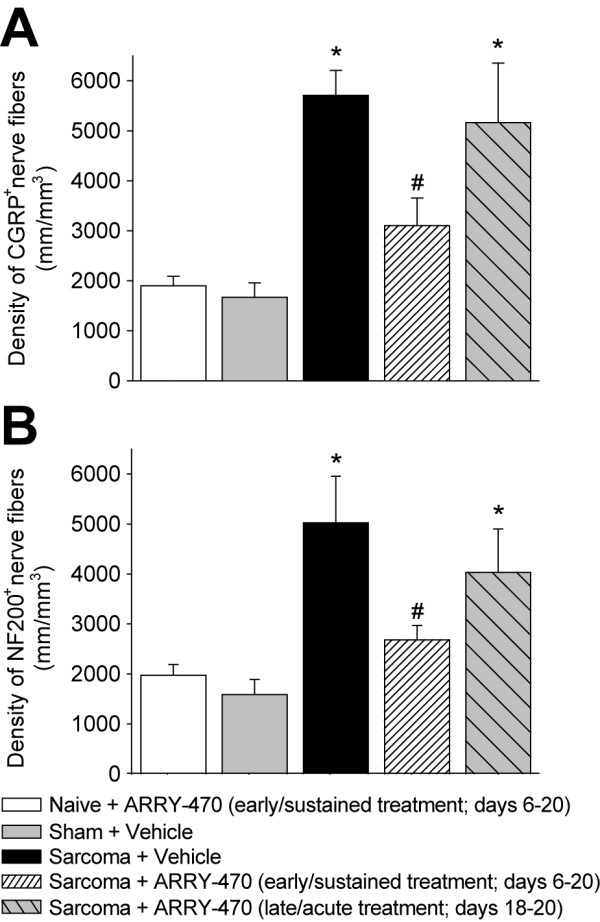
**Preventive administration of ARRY-470 therapy reduces sarcoma-induced nerve sprouting of CGRP^+ ^and NF200^+ ^nerve fibers**. At day 20 post cell injection, the density of CGRP^+ ^(A), NF200^+ ^(B) nerve fibers is significantly greater in sarcoma + vehicle-treated mice compared to sham + vehicle-treated mice. This tumor-induced nerve sprouting is significantly attenuated by early/sustained administration of ARRY-470 (30 mg/kg; p.o., BID) initiated at day 6 and maintained through day 20 post tumor cell injection), but not by late/acute (30 mg/kg; p.o., BID given at day 18 and maintained through day 20 post tumor cell injection) administration of ARRY-470. Nerve fiber density was determined by measuring the total length of nerve fibers per unit volume in the periosteum. Bars represent the mean ± SEM. *p < 0.05 vs. sham + vehicle, # p < 0.05 vs sarcoma + vehicle. The number of animals was n = 8 for sham, n = 9 for sarcoma + vehicle, n = 9 for sarcoma + early/sustained ARRY-470, and n = 7 for sarcoma + late/acute ARRY-470.

Early/sustained treatment with ARRY-470 (given twice daily from 6 to 20 days post tumor injection) largely prevented the sprouting of CGRP^+ ^(Figure 6C, Figure 7A) and NF200^+ ^(Figure 6F, Figure 7B) nerve fibers. Interestingly, the attenuation of this sprouting was observed only with the early/sustained ARRY-470 administration, and not with the late/acute administration (Figure 7A, B). Early/sustained administration of ARRY-470 similarly resulted in a marked decrease in the formation of neuroma-like structures of CGRP^+ ^and NF200^+ ^nerve fibers. Importantly, early/sustained administration of ARRY-470 did not affect the organization or density of CGRP^+ ^or NF200^+ ^fibers in the contralateral, non-tumor bearing bones compared to sham mice (data not shown).

In addition, the effects of ARRY-470 therapy on tumor growth and bone destruction were examined at day 14 and 20 post tumor injection, respectively. Treatment of sarcoma-injected mice with ARRY-470 at days 6-20 post tumor injection resulted in no reduction in tumor growth (100 ± 0 of intramedullary space (Additional file 1 Figure S1B) and no significant change in bone resorption (2.6 ± 0.24; as compared to sarcoma + vehicle animals (2.8 ± 0.14) (Additional file 1 Figure S1E).

## Discussion

### Blockade of Trks and skeletal pain

In the present study we show that early/sustained administration of a Trk inhibitor significantly inhibited sprouting and neuroma formation by sensory nerve fibers and reduced bone cancer pain-related behaviors by 50-60%. As the Trk inhibitor has a 50:1 plasma to CSF ratio, the anti-hyperalgesic actions of the inhibitor would appear to occur primarily outside the blood brain barrier. Previous reports have demonstrated that following peripheral inflammation and tissue injury, a variety of inflammatory, immune and stromal cells upregulate the expression of NGF, brain-derived neurotrophic factor (BDNF) and neurotrophin-3 (NT-3) whose cognate receptors are TrkA, TrkB and TrkC respectively. Many studies have shown that peripheral NGF can drive pain and have suggested that NGF and perhaps peripherally released BDNF and NT-3 may play a role in modulating pain [14,15]. As the present results show that blockade of all three Trks reduces bone cancer pain, a key question is which neurotrophins and Trks are most likely the major contributors to the generation and maintenance of bone cancer pain.

Previous results have demonstrated that in the adult NGF can directly activate and sensitize sensory neurons involved in the conduction of pain originating from the skin [16,17], viscera and skeleton. NGF is thought to excite and sensitize sensory neurons by binding to its cognate receptor TrkA which is expressed by a subpopulation of mostly unmyelinated and thinly myelinated sensory neurons [18]. NGF binding to TrkA has been shown to directly activate TrkA-expressing nociceptors *in vivo *and *in vitro *and that binding of NGF to TrkA directly lowers the threshold for depolarization in these neurons [19,20]. Additionally, NGF has been shown to modulate and/or sensitize a variety of neurotransmitters, receptors, ion channels and structural molecules expressed by nociceptors [17]. It has also been shown that NGF lowers the threshold and enhances the response of nociceptors to mechanical stimuli [21], suggesting that NGF activation of TrkA may play a role in activating/sensitizing mechanotransducers expressed by sensory nerve fibers. The NGF produced by target tissues or tumor cells activates TrkA receptors expressed on the terminals of C-fibers [18,22] presumably including those innervating the skeleton. Whether the Trk inhibitor used here is exerting its effect by interfering with the retrograde signal (the internalized NGF/TrkA complex) that exerts transcriptional control in the neuronal cell body or by local modulation at the nociceptor terminal is not clear. However, in a model of bone fracture pain, with Trk inhibition at 2 days post fracture, where nerve sprouting has not yet occurred, the full analgesic effect is achieved 6-8 hours following acute administration [23]. These data suggest that local modulation of nerve fibers must be involved, as transport of the NGF/TrkA from the nerve terminals in the femoral fracture site to the cell bodies of sensory neurons that innervate the femur (which are located in the L1-L3 ganglia) would be expected to take significantly longer than 8 hours [24].

While there is strong evidence that TrkB receptors expressed by post-synaptic spinal cord neurons play a significant role in pain transmission [17] there is significantly less agreement about the role of TrkB expressed by sensory neurons in driving pain. Previous reports have suggested that TrkB receptors are expressed by a subpopulation of the DRG, nodose and trigeminal neurons and their terminals in the spinal dorsal horn and trigeminal nucleus [25-27] and that peripheral inflammation in some tissues results in an increase in BDNF levels [16]. Interestingly, in pancreatitis BDNF content was reported to be correlated with pain intensity [28] and exogenous application of BDNF has been shown to excite and sensitize some cutaneous nociceptive terminals [29] (apparently via TrkB). However, the effects of BDNF on TrkB expressing sensory neurons in driving any type of chronic pain remain poorly understood [17]. Similarly, there is relatively little evidence to suggest that peripheral TrkC receptors expressed by myelinated nerve fibers play a significant role in the generation and maintenance of pain in the adult. Thus, while local injection of NT-3 has been reported to induce mild pain at the injection site [30], other reports suggest that NT-3 does not sensitize nociceptive primary afferent fibers [20] and appears to be anti-nociceptive in some pain models such as complete Freund's adjuvant-induced skin inflammation [31].

The above results, together with the present data demonstrating that the analgesic efficacy of the Trk inhibitor in blocking bone cancer pain is similar to that of anti-NGF sequestering therapy, suggest that TrkA plays the prominent role in driving bone cancer pain [32]. One unique aspect of the sensory innervation of bone, which may partially explain why Trk inhibition is effective in relieving skeletal pain, is that the majority of C-fibers that innervate the bone are CGRP-expressing fibers, and nearly all CGRP^+ ^fibers co-express TrkA [18,22]. Thus, most C-fibers that innervate both human [33] and rodent [34] vertebral discs and bone [35] appear to be CGRP/TrkA expressing fibers and few unmyelinated non-peptidergic IB4/RET^+ ^nerve fibers are present in these tissues [10,35]. Thus, since bone appears to lack the redundancy of the C-fiber non-peptidergic IB4/RET^+ ^nerve fibers that are present in skin [10], blocking TrkA activation may be particularly efficacious in relieving bone pain vs. skin pain.

### Trks and their involvement in the development of nerve sprouting and neuroma formation

Although Trks clearly play an essential role in the growth and survival of sensory neurons in the developing animal [36,37], much less is known about the role the Trks play in the maintenance and survival of adult sensory neurons. In the adult, neurotrophins appear to be expressed in most tissues at very low levels, whereas the levels of NGF (and in some tissues BDNF and NT-3) are dramatically up-regulated by inflammation and/or injury [14,16]. However, it has been shown that chronic NGF deprivation (by exogenous administration of an anti-NGF polyclonal antibody or autoimmunization to NGF) results in a modest hypoalgesia, where animals are less sensitive to some thermal and algogenic stimuli [38]. Whether this hypoalgesic effect observed in rats with polyclonal antibodies or autoimmunization will also be observed in humans treated with Trk inhibitors is unclear as is how much endogenous NGF, BDNF or NT-3 is required to maintain normal sensory nerve function in the adult.

The present studies suggest that similar to the tumor, sensory nerve fibers undergo a highly pathological and active reorganization as tumor cells invade the bone. Thus, as the tumor and associated stromal cells invade the bone, there is significant sprouting by CGRP^+ ^and NF200^+ ^sensory nerve fibers and these sensory nerve fibers are intermingled among the tumor/stromal cells that have invaded and remodeled the bone. These newly sprouted CGRP^+ ^and NF200^+ ^nerve fibers have a very dense and highly disorganized morphology that is never observed in the normal bone. In addition to the sprouting of nerve fibers, in approximately 1 out of 2 tumor-bearing bones we observe the appearance of neuroma-like structures that looked very similar to neuromas that have been described in both animals and humans following traumatic nerve injury. These structures appear as a disordered mass of CGRP^+ ^and NF200^+ ^blind ending axons that generally run parallel to each other and have an interlacing or whirling morphology [39,40]. It should be emphasized that we have never observed these neuroma-like structures in the sham vehicle-treated or naïve bones. However, these data would agree with previous findings suggesting that NGF is involved in neuroma formation and when provided with the appropriate trophic factor, sensory nerve fibers can grow at a remarkable pace, sprouting several millimeters a day [41].

Previous studies have shown that injury to peripheral nerves associated with trauma, amputation, compression, or surgery can lead to painful neuromas [13,42,43], which have a morphology similar to the neuroma-like structures observed in the tumor-bearing mouse bones. In humans, these non-malignant neuromas frequently cause chronic and severe pain [13,43] and can produce spontaneous ectopic discharges [44,45] in part by up-regulation of sodium channels [43,46]. Problematically, painful neuromas can be largely refractory to current medical treatment [43]. It is not currently known whether there is up-regulation of sodium channels and spontaneous discharge by these neuroma-like structures in the tumor bearing mouse bone. However, movement may not be required for these ectopic discharges to occur suggesting that this mechanism is a possible explanation for spontaneous breakthrough pain.

## Conclusions

The present study shows that early/sustained administration of a small molecule Trk inhibitor attenuates sensory nerve fiber sprouting, neuroma formation, and bone cancer pain-related behaviors. Previous studies have shown that inappropriate remodeling of sensory nerve fibers, whether through sprouting or neuroma-like formation, can give rise to hyperalgesia, allodynia, and spontaneous ectopic discharges that are perceived as highly painful in humans [13,43,47]. The present data suggest that early/sustained administration of therapies that block the NGF/TrkA axis may be more effective than late administration in reducing the ectopic sprouting as well as cancer pain.

## Methods

### Animals

Experiments were performed on a total of 120 adult male C3H/HeJ mice (Jackson Laboratories, Bar Harbor, ME), initially at 8 weeks of age, weighing 20-25 g. The mice were housed in accordance with the National Institutes of Health guidelines under specific pathogen free conditions in autoclaved cages maintained at 22°C with a 12-hour alternating light and dark cycle and were given autoclaved food and water ad libitum. All procedures were approved by the Institutional Animal Care and Use Committee at the Minneapolis VA Medical Center.

### Surgical procedure for implantation of cancer cells

Osteolytic murine sarcoma cells were obtained (NCTC 2472, ATCC, Rockville, MD), stably transfected with green fluorescent protein, maintained and surgically implanted into the mouse femur as previously described [32] (Additional file 1 Figure S1).

### Treatment with Trk inhibitor (ARRY-470) therapy

The Trk inhibitor (ARRY-470; Array BioPharma, Boulder, CO) is a potent inhibitor of the tropomyosin kinase family of neurotrophin receptors, demonstrating nanomolar cellular inhibition of TrkA (6.5 nM), TrkB (8.1 nM), and TrkC (10.6 nM) and a high level of selectivity over a panel of kinases run at the ATP K_m _at 1.0 uM and non-kinase receptors [48](Additional file 1 Table S1 and S2). At doses of 10-100 mg/kg ARRY-470 reaches high concentrations in plasma and peripheral tissues, while the brain concentrations remain negligible, suggesting very limited crossing of the blood brain barrier [49]. ARRY-470 at a dose of 30 mg/kg in a mouse xenograft model derived from HEK cells constitutively expressing active human TrkA showed >90% inhibition of phosphorylated TrkA at 1 hour and >70% inhibition over a 12 hour time course [49]. Additionally, results from previous studies have demonstrated that administration of 30 mg/kg ARRY-470 significantly reduced thermal hyperalgesia and mechanical allodynia in a rat CFA model of inflammation [49].

To evaluate the effect of early vs. late dosing of a Trk inhibitor on pain-related behaviors, neurochemical changes and disease progression, treatment with ARRY-470 (30 mg/kg, p.o., bid) was initiated either when cancer-induced pain behaviors became evident (day 6-20 post-sarcoma injection) or after significant disease progression (day 18-20 post-sarcoma injection).

### Behavioral measures of cancer pain

Pain behavior analysis was performed as previously described [32] (Additional file 1 Methods) and used to evaluate of the analgesic efficacy of ARRY-470 (30 mg/kg, p.o.) in attenuating bone cancer-related pain behavior. Assessment of bone cancer pain-related behaviors, including spontaneous guarding and flinching of the hind limb, was performed on days 8, 10, 12, 14, 16, 18 and 20 following tumor cell or sham inoculation. Behavioral analyses were performed within 30-60 minutes of administration of the drug or vehicle (Labrafac;polyglycolyzed glyceride).

### Immunohistochemistry

Mice were sacrificed by carbon dioxide asphyxiation, delivered using a compressed gas cylinder, at day 20 post sarcoma injection and perfused intracardially with 20 ml of 0.1 M phosphate buffered saline (PBS, pH = 7.4 at 4°C) followed by 30 ml of 4% formaldehyde/12.5% picric acid solution in 0.1 M PBS (pH = 6.9 at 4°C). Ipsilateral and contralateral femurs were harvested following perfusion and post-fixed for at least 12 hours in the perfusion fixative.

To qualitatively assess the tumor-induced changes in the density and morphology of sensory nerve fibers that innervate the periosteum, whole mount preparations were processed on 20 bones according to the our previously published procedures [32]. The size of the periosteal whole mount preparation and its attached thin muscle layer used for immunohistochemistry was approximately: width = 6 mm; length = 6 mm; thickness = 0.5 mm.

For quantification, frozen sections were used as the cross sections allow visualization of the bone's landmarks (such as the growth plate), which enable the observer to locate the same anatomical area when quantifying changes in nerve fibers in different animals. The process of post-fixing, decalcification and sectioning of the femurs was performed as previously described [32].

Periosteum whole mounts and frozen sections were processed according to previously published procedures [32] using a marker of primary afferent sensory neurons (polyclonal rabbit anti-rat CGRP; 1:10,000; Sigma Chemical Co., St. Louis, MO; Catalog number C8198) and a marker of myelinated primary afferent sensory nerve fibers (chicken anti-neurofilament 200Kd; NF200, 1:1000; Chemicon, Temecula, CA; Catalog number AB5539). GFP expression levels did not require amplification to quantify tumor progression.

### Quantification of nerve fiber density, sprouting and neuroma formation

The density of CGRP^+ ^and NF200^+ ^nerve fibers in the periosteum following treatment with vehicle or ARRY-470 was determined by capturing images of these nerve fibers in periosteal frozen sections using an Olympus Fluoview FV1000 laser scanning confocal imaging system (Olympus America Inc, Melville, NY, software v. 5.0). Approximately 30 separate, 20 μm thick frozen sections were obtained from each femur. Three images were obtained for each marker (200× magnification) and each image was acquired within 2 mm distal from the proximal femoral growth plate, with images taken from different sections at least 100 μm apart. The average area of periosteum that was analyzed was 620 μm (length), 70 μm (width), 20 μm (depth). The Z-stacked images were analyzed with Image-Pro Plus v. 6.0 (Media Cybernetics) and nerve fibers were manually traced to determine the length of nerve fibers. Nerve sprouting was reported as density of nerve fibers per volume of periosteum [32].

To quantify the extent of formation of neuroma-like structures, frozen sections were examined with a fluorescent microscope and these structures were manually counted and totaled from the entire 20 um thick section. Three different sections, each at least 100 um apart, were evaluated per animal. A neuroma-like structure was defined as i.) a disordered mass of blind ending axons (CGRP^+ ^or NF200^+^) that has an interlacing and/or whirling morphology, ii.) a structure with a size of more than 10 individual axons that is at least 20 μm thick and 70 μm long, and iii.) a structure which is never observed in the periosteum of normal bone [39,40].

### Quantification of tumor growth and **tumor-induced bone destruction**

Images of sections from tumor-bearing femurs were acquired and the total area of intramedullary space and the percent of intramedullary space occupied by tumor cells were calculated using Image Pro Plus v6.0 software (Media Cybernetics, Silver Spring, MD) as previously described [9]. Area of intramedullary space occupied by tumor cells is presented as a percentage of total intramedullary area. Radiograph images of the medial-lateral plane of both bones were used to evaluate tumor-induced bone destruction as previously described [10] (Additional file 1 Figure S1).

### Statistics

A one-way ANOVA was used to compare behavioral results and immunohistochemical measures between the experimental groups. For multiple comparisons, the Fisher's PLSD (Protected Least Significant Difference) post hoc test was used. Significance level was set at P < 0.05. In all cases, the investigator responsible for behavioral testing, plotting, measuring, and counting was blind to the experimental situation of each animal.

## Competing interests

The authors declare that they have no competing interests.

## Authors' contributions

JRG participated in the design of the study, analysis and interpretation of the data, and drafted the manuscript. KTF participated in the analysis and interpretation of the animal behavior data. JMJA participated in the interpretation and analysis of the immunohistochemical data and contributed to the design of the study. WGM participated in the interpretation and analysis of the immunohistochemical data. APB participated in the interpretation and analysis of the μCT data. MAK provided the statistical analysis. PWM conceived of the study, provided analysis and interpretation of the data and significantly contributed to drafting the manuscript. All authors read and approved the final manuscript.

## Supplementary Material

Additional file 1 **Figure S1. Confocal and uCT images of tumor growth and bone remodeling at day 20 post-tumor cell injection**. Sarcoma+vehicle and sarcoma+ARRY-470 femurs are immunoreactive for GFP, however, no significant difference in tumor growth or tumor induced bone destruction was observed. In addition, sham animals treated with vehicle show no radiographically apparent bone destruction at day 20, whereas sarcoma+vehicle treated animals show a transition from the radio-opaque bone tissue to a radiolucent appearance by day 20. **Table S1. A broad radiometric protein screen to determine the selectivity of a Trk inhibitor (ARRY-470) vs. a diverse panel of kinases**. Trk inhibitor ARRY-470 is > 100 fold selective when tested against a diverse panel of 229 radiometric protein kinases. **Table S2. A broad radioligand screen to determine the selectivity of a Trk inhibitor (ARRY-470) vs. a diverse panel of receptors, channels, and transporters**. Trk inhibitor ARRY-470 is > 1000 fold selective when tested against a diverse panel of receptors, channels, and transporters. The Trk inhibitor ARRY-470 does not show any significant inhibition against this panel of receptors. In contrast, ARRY-470 inhibits Trks A, B, C with IC50 < = 11 nM. Click here for file
