# Supplementary material for: Administration of a tropomyosin receptor kinase inhibitor attenuates sarcoma-induced nerve sprouting, neuroma formation and bone cancer pain
Source: Mol Pain. 2010 Dec 7;6:87. doi: 10.1186/1744-8069-6-87 (PMC3004846; doi:10.1186/1744-8069-6-87)
Supplement: Additional file 1 — Figure S1. Confocal and uCT images of tumor growth and bone remodeling at day 20 post-tumor cell injection. Sarcoma+vehicle and sarcoma+ARRY-470 femurs are immunoreactive for GFP, however, no significant difference in tumor growth or tumor induced bone destruction was observed. In addition, sham animals treated with vehicle show no radiographically apparent bone destruction at day 20, whereas sarcoma+vehicle treated animals show a transition from the radio-opaque bone tissue to a radiolucent appearance by day 20. Table S1. A broad radiometric protein screen to determine the selectivity of a Trk inhibitor (ARRY-470) vs. a diverse panel of kinases. Trk inhibitor ARRY-470 is > 100 fold selective when tested against a diverse panel of 229 radiometric protein kinases. Table S2. A broad radioligand screen to determine the selectivity of a Trk inhibitor (ARRY-470) vs. a diverse panel of receptors, channels, and transporters. Trk inhibitor ARRY-470 is > 1000 fold selective when tested against a diverse panel of receptors, channels, and transporters. The Trk inhibitor ARRY-470 does not show any significant inhibition against this panel of receptors. In contrast, ARRY-470 inhibits Trks A, B, C with IC50 < = 11 nM. [file 1744-8069-6-87-S1.DOC]

**ADDITIONAL FILES**


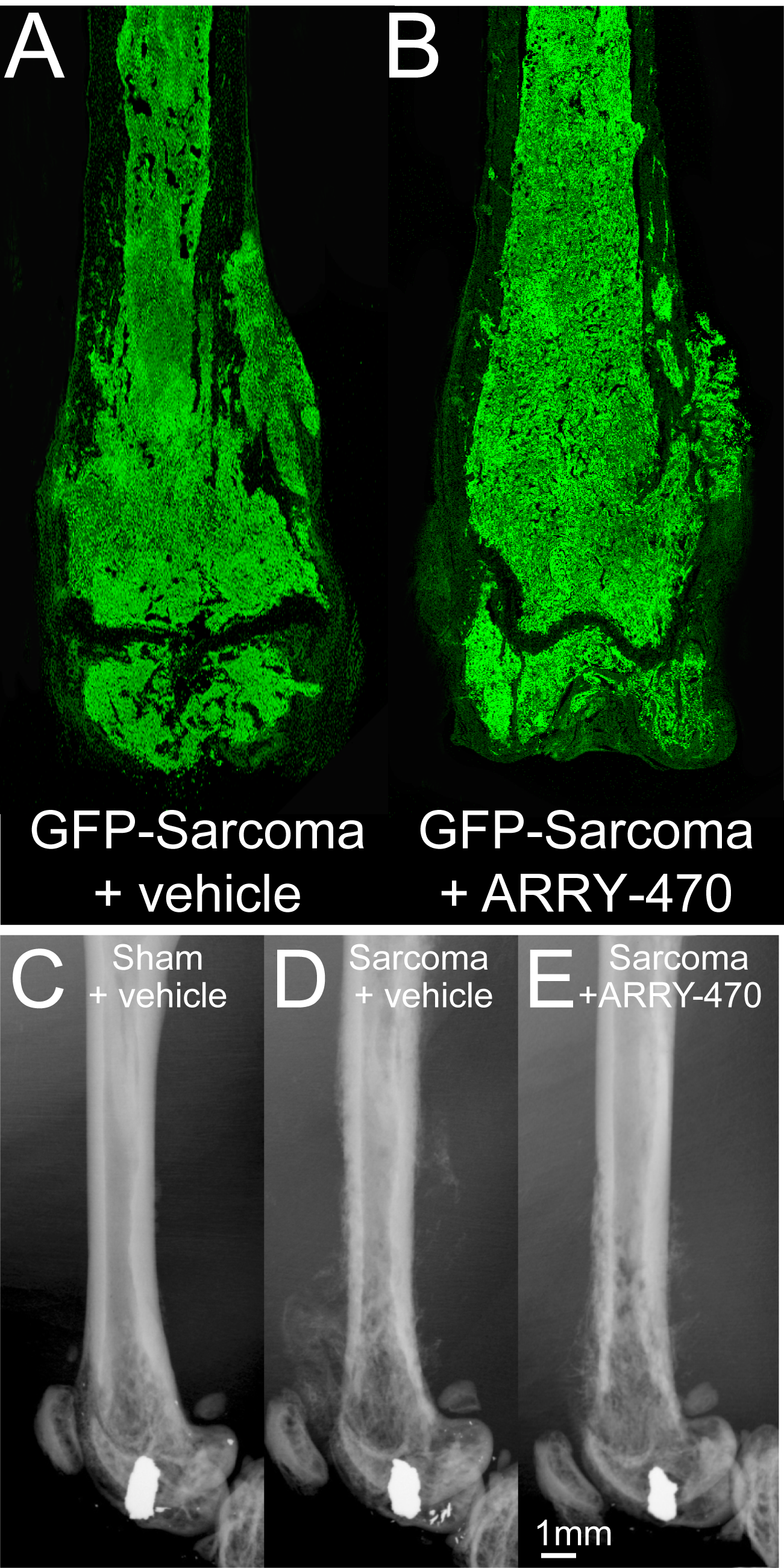


Additional Figure S1.

**ARRY-470 therapy had no significant effect on tumor growth and bone remodeling at day 20 post tumor cell injection.** GFP transfected tumor cells injected into the femurs were immunostained with an antibody raised against GFP. Sarcoma+vehicle and sarcoma+ARRY-470 femurs are immunoreactive for GFP (A, B); however, no significant difference in tumor growth or tumor induced bone destruction was observed. Sham animals treated with vehicle (n=8, C) show no radiographically apparent bone destruction at day 20, whereas sarcoma+vehicle treated animals (n=13) show a transition from the radio-opaque bone tissue to a radiolucent appearance by day 20 (D). Sarcoma+ARRY-470 (E) animals (n=8) present the same pattern and extent of bone destruction as sarcoma+vehicle treated animals. Scale bars: A–B 4 mm; C-E 1 mm.

Trk Inhibitor ARRY-470 is >100 Fold Selective vs. a Diverse Panel of Kinases.

Additional Table S1.

Trk inhibitor ARRY-470 is selective when tested against a diverse panel of kinases.

Briefly, KinaseProfiler assay (Millipore/Upstate) consists of 229 radiometric protein kinase assays. Each specific kinase is incubated with a MOPS buffer, peptide specific to the kinase assay, MgAcetate and gamma33P-ATP.  The reaction is initiated by the addition of the MgATP mix.  After incubation at room temperature the reaction is stopped using a phosphoric acid solution.  An aliquot of the reaction is then spotted on a filter, washed with phosphoric acid, then methanol once prior to drying and scintillation counting. For further information see www.millipore.com.

Trk Inhibitor ARRY-470 is >1000 Fold Selective vs. a Diverse Panel of Receptors.

Additional Table S2.

Trk inhibitor ARRY-470 specifically inhibits Trks in a broad radioligand screen of receptors, channels and transporters. Briefly each assay is run in duplicate at 10 µM ARRY-470. The radioligand binding assay source is either human recombinant cells or mouse, guinea pig or rat tissue specific for each assay. The radiolabeled ligand is specific for each receptor and incubations are performed in a neutral pH buffer with compound or a non specific ligand for a control. Incubation time and conditions vary for each assay with significance being defined as > 50% stimulation or inhibition of ligand binding. The Trk inhibitor ARRY-470 does not show any significant inhibition against this panel of receptors. In contrast, ARRY-470 inhibits Trks A, B, C with IC50<= 11nM. For further information see www.ricerca.com.

**Methods**

***Surgical procedure and implantation of cancer cells***

Osteolytic murine sarcoma cells were obtained (NCTC 2472, ATCC, Rockville, MD), stably transfected with green fluorescent protein and maintained as previously de- scribed.15 Following induction of general anesthesia with ketamine:xylazine (75 mg/kg, intramuscularly), a superficial incision was performed in skin overlying the patella to expose the condyles of the distal femur. A 30-gauge needle was inserted at the level of the intercondylar notch and into the medullary canal to create an initial core pathway. A 0.5 mm depression was then made using a 1/2 round bur in a pneumatic dental high-speed handpiece to serve as mechanical retention for an amalgam plug. Hanks balanced salt solution (HBSS, 20µl, Cellgro, Manassas, VA) or HBSS containing 105 2472 osteolytic sarcoma cells (20µl) was injected into the intramedullary cavity using a syringe. In order to prevent reflux of cells outside the bone, obturation of the injection site with dental grade amalgam (Dentsply) was performed using an endodontic messing gun (Union Broach) followed by copious irrigation with sterile filtered water. Wound closure was achieved using a single 7-mm auto wound clip (Becton Dickinson, Sparks, MD).

***Behavioral measures of cancer pain***

To evaluate the effect of early vs. late dosing of a Trk inhibitor on pain-related behaviors, ARRY-470 (30 mg/kg, p.o., bid) was initiated either when cancer-induced pain behaviors became evident (day 6-20 post-sarcoma injection) or after significant disease progression (day 18-20 post-sarcoma injection). Mice were behaviorally tested on day 8, 10, 12, 14, 16, 18 and 20 following tumor cell or sham inoculation as previously described (9). Animals were observed over a standardized two-minute period each day to characterize the spontaneous bone cancer pain behaviors. Briefly, mice were placed in a clear plastic observation box with a wire mesh floor and allowed to habituate for a period of 15 min. Following acclimation, the number of flinches and time spent guarding the tumor-bearing limb were recorded as measures of ongoing pain, as these measures mirror patients in a clinical setting with bone cancer who protect or suspend their tumor-bearing limb. Flinches were defined as the number of times the animal raised its hindpaw aloft while not ambulatory. Guarding was defined as the time the hindpaw was held aloft while ambulatory. The investigator was blinded as to the experimental condition of the animals.

***Radiographic Analysis of Tumor-Induced Bone Destruction***

Following behavioral analysis, mice were lightly anesthetized (80mg/kg ketamine/4mg/kg xylazine, i.p.) and digital radiographs (MX20 DC12, Faxitron X-Ray) of lower extremities were obtained. Radiograph images of the medial-lateral plane of both bones were used to evaluate tumor-induced bone destruction as previously described (15). Radiographs of tumor-bearing femora, were used to evaluate bone destruction and were assigned scores of 0–4: 0, normal bone with no signs of destruction; 1, small radiolucent lesions indicative of bone destruction (one to three lesions); 2, increased number of lesions (three to six lesions) and loss of medullary bone; 3, loss of medullary bone and erosion of cortical bone; 4, full-thickness unicortical bone loss. Analysis was performed in a blinded fashion.

***Micro-Computed Tomographic Images of Normal vs. Tumor Bone Bearing Destruction***

In order to image disease progression and cancer-induced changes in mineralized bone, femurs were analyzed with an eXplore Locus SP micro-computed tomographer (GE Healthcare, London, Ontario, Canada). The system uses a microfocal X-ray tube providing an 8-mm focal spot. This conebeam μCT scanner uses a 2300 × 2300 CCD detector with current and voltage set at 80 µA and 80 KVp, respectively. Specimens were scanned in 1080 views through 360° with a 2100 ms integration time. The specimen is loaded on a turntable that can be shifted automatically in the axial direction. After a data acquisition time set at 300 milliseconds, the turntable with the specimen was rotated by 1􏰁and a new data acquisition process was performed. Scans were then reconstructed at 16-µm3 resolution using Reconstruction Utility software (GE Healthcare). A standard convolution back projection procedure was used to reconstruct the CT images in 1,024 x 1,024 pixel matrices.
